# IL-13 Induces YY1 through the AKT Pathway in Lung Fibroblasts

**DOI:** 10.1371/journal.pone.0119039

**Published:** 2015-03-16

**Authors:** Jia Guo, Hongwei Yao, Xin Lin, Haodong Xu, David Dean, Zhou Zhu, Gang Liu, Patricia Sime

**Affiliations:** 1 Department of Medicine, University of Rochester Medical School, Rochester, New York, United States of America; 2 Department of Environmental Medicine, University of Rochester Medical Center, Rochester, New York, United States of America; 3 Department of Pediatrics, University of Rochester; Rochester, New York, United States of America; 4 Pathology and Laboratory Medicine, David Geffen School of Medicine, UCLA, Los Angeles, California, United States of America; 5 Department of Allergy and Clinic Immunology, Yale University, New Haven, Connecticut, United States of America; 6 Department of medicine, Pulmonary and critical care, University of Alabama, Birmingham, Alabama, United States of America; Medical University of South Carolina, UNITED STATES

## Abstract

A key feature of lung fibrosis is the accumulation of myofibroblasts. Interleukin 13 (IL-13) is a pro-fibrotic mediator that directly and indirectly influences the activation of myofibroblasts. Transforming growth factor beta (TGF-β) promotes the differentiation of fibroblasts into myofibroblasts, and can be regulated by IL-13. However, IL-13’s downstream signaling pathways are not completely understood. We previously reported that the transcription factor Yin Yang 1 (YY1) is upregulated in fibroblasts treated with TGF-β and in the lungs of mice and patients with pulmonary fibrosis. Moreover, YY1 directly regulates collagen and alpha smooth muscle actin (α-SMA) expression in fibroblasts. However, it is not known if IL-13 regulates fibroblast activation through YY1 expression. We hypothesize that IL-13 up-regulates YY1 expression through regulation of AKT activation, leading to fibroblast activation. In this study we found that YY1 was upregulated by IL-13 in lung fibroblasts in a dose- and time-dependent manner, resulting in increased α-SMA. Conversely, knockdown of YY1 blocked IL-13-induced α-SMA expression in fibroblasts. Furthermore, AKT phosphorylation was increased in fibroblasts treated with IL-13, and AKT overexpression upregulated YY1, whereas blockade of AKT phosphorylation suppressed the induction of YY1 by IL-13 *in vitro*. *In vivo* YY1 was upregulated in fibrotic lungs from CC10-IL-13 transgenic mice compared to that from wild-type littermates, which was associated with increased AKT phosphorylation. Taken together, these findings demonstrate that IL-13 is a potent stimulator and activator of fibroblasts, at least in part, through AKT-mediated YY1 activation.

## Introduction

Idiopathic pulmonary fibrosis (IPF) is characterized by the accumulation and activation of fibroblasts and myofibroblasts, deposition of extracellular matrix (ECM) proteins, such as collagen, as well as distortion of normal tissue architecture [1]. This serious lung disorder presents major clinical challenges because there are few effective therapeutic agents for preventing or reversing lung fibrosis [2], and the precise molecular mechanisms underlying persistent fibroblast activation remains poorly understood.

Myofibroblasts, derived from fibroblasts via the activity of TGF-β and other stimuli [3], are recognized as major effector cells in pulmonary fibrosis. They are characterized by the increased expression of α-smooth muscle actin (α-SMA), enhanced proliferation [4] and resistance to apoptosis[5]. Their accumulation is promoted by the profibrotic cytokine TGF-β.

A number of other cytokines have also been implicated in fibrosis. Among them, IL-13 is thought to be important cytokine in the pathogenesis of asthma, and more recently has been shown to play a pivotal role in a number of fibrotic diseases including hepatic and pulmonary fibrosis, and nodular sclerosing Hodgkin’s disease [6,7,8,9,10,11,12]. It has been shown that IL-13 induces lung fibrosis by selectively stimulating TGF-β [13,14]. During chronic inflammation, IL-13 is induced concomitantly with activation of its downstream signaling via an IL-13 receptor, IL-13 Rα2 [13,15]. This receptor signaling induces production of TGF-β, which can induce IGF and Egr-1 expression in myofibroblasts [16,17,18]. IL-13 also binds to IL-4 receptor alpha and IL-13 receptors alpha1, leading to activation of the canonical STAT6-signaling pathway. A separate study demonstrates that IL-13 can activate phosphatidylinositol 3-kinase (PI3K)/AKT signals in endothelial cells [19]. Relevant to fibrogenesis, inhibition of PI3K and protein kinase B (PKB)/AKT diminishes TGF-β–induced fibrosis [11], whereas activation of PKB/AKT by TGF-β mediates stable induction of myofibroblast differentiation and survival [20]. Furthermore, TGF-β is able to activate pro-survival signaling pathways involving AKT and FAK to induce an apoptosis-resistant myofibroblast phenotype [21]. However, it is not known whether PI3K/AKT signaling is involved in IL-13-induced lung fibrosis.

Yin Yang 1 (YY1) is a transcription factor that plays an important role in cell cycle regulation, proliferation and differentiation. YY1 inhibition negatively regulates Fas and DR5 expression, and TNF-related apoptosis [22]. We previously demonstrated that YY1 is up-regulated by TGF-β through NF-κB directly binding to the YY1 promoter in lung fibroblasts [23]. In addition, YY1 can directly upregulate α-SMA and collagen expression, and YY1 knockdown protects against lung fibrosis by decreasing collagen and α-SMA [23]. However, it is unclear whether both YY1 and PI3K/AKT are involved in IL-13-dependent pro-fibrotic pathways in the lungs. Therefore, we hypothesize that IL-13 induces lung fibrosis by activation of YY1 through an AKT pathway. To test this hypothesis, we employed both pharmacological and genetic approaches to manipulate the expression of IL-13 and AKT in human lung fibroblasts and mouse lungs. Targeting these pro-survival signaling pathways may provide an effective strategy to control fibroblast accumulation in the years ahead [24,25,26].

## Materials and Methods

### Ethics statement

All animals were housed under specific pathogen-free conditions in an American Association for the Accreditation of Laboratory Animal Care-approved facility in accordance with the standards established by the United States Animal Welfare Act, as set forth by the National Institutes of Health guidelines. Mice were given ad libitum access to food and water. The protocol was approved by the Institutional Animal Care and Use Committee (University Committee on Animal Resources (UCAR) protocol 2006-086/100473 of the University of Rochester. All animal experiments described in this report were reviewed and approved by the University Committee on Animal Resources (IACUC) at the University of Rochester Medical Center (UCAR#: A-3292-01).

### Animal and cell lines

Mice were generated from a mating colony by intercrossing heterozygous sires of B6-129 mixed background (129S7/AB2.2 × C57BL6). CC10-driven IL-13 transgenic/overexpressing (CC10-IL-13) mice were obtained from Z. Zhu (Johns Hopkins University). Wild type C57BL/6 mice were obtained at 5 weeks of age and housed 5 per microisolator cage in a vivarium housing room on a 12-hour light/dark cycle at the University of Rochester Medical Center. CC10-driven IL-13 transgenic/overexpressing (CC10-IL-13) mice (C57BL/6 background) were obtained from Z. Zhu (Johns Hopkins University). The generation of the transgenic mouse was described previously [13]. Male CC10-IL-13 mice and wild type littermates (5 weeks old) were used for the experiments. The MRC-5 human fibroblasts and LL97A fibroblasts were purchased from ATCC.

### Histopathological and immunohistochemical examination

Lungs were fixed with 10% buffered formalin and embedded in paraffin. H&E and Masson’s Trichrome stains were performed for the analysis of pathological changes. Immunohistochemical staining for α-SMA, FSP1 and YY1 were performed as described previously (21). FSP1 antibody was purchased from NewMarkers (Cat. #RB-1804-A0). Briefly, lung sections were deparaffinized, incubated with 10 mM of hot citrate buffer (pH 6.0) to retrieve antigen, blocked, and stained with primary antibodies overnight at 4°C. After application of the corresponding secondary antibody, streptavidin conjugated-horeseradish peroxidase (HRP-Streptavidin) was applied, and staining was visualized with NovaRed or DAB substrate (Vector Laboratories). Slides were counterstained with hematoxylin for determination of FSP1 and α-SMA expression.

### Quantitative PCR

MRC5 fibroblast cells were pretreated with or without wortmannin (10 μM) for 1 h followed by treatment with or without IL-13 (30 ng/ml) for 12 h. Total RNA from MRC5 cells was extracted using TRIzol reagent (Invitrogen). Extractions were quantified with Nanoreader. Subsequently, 1 μg of total RNA was reverse-transcribed using oligo dT as a primer. Quantitative PCR was performed using an iQ5 Bio-Rad device. Data was analyzed by the 2^−^∆^(^∆^C^_T_^)^ method and normalized with GAPDH housekeeping gene.

### Immunofluorescent staining

After MRC5 cells were stimulated by IL-13 (30 ng/ml) for 12 h or LL97A cells were transfected with YY1 shRNA, and plated in four-well chamber slides supplemented in DMEM with 10% FBS at 37°C for two days, cells were fixed with 4% paraformaldehyde, permeabilized with 0.1% PBS-Triton-100, blocked with protein block serum-free solution (DAKO Cytomation), and incubated with an anti-α-SMA antibody (1A4, DAKO Cytomation) for 1h at room temperature. The cells were then incubated with a secondary antibody (goat anti-mouse Alexa Fluor 488, Invitrogen) for 1h at room temperature. Anti-YY1 antibody (H-414, Santa Cruz) incubation was performed at 4°C overnight. The secondary antibody (goat anti-rabbit Texas Red-X, Invitrogen) was used for 1h at room temperature. The stained cells were embedded in a mounting medium with DAPI (Invitrogen) and examined with a Zeiss fluorescence microscope.

### Luciferase assay and construction of the YY1 and α-SMA luciferase reporter

Plasmid containing only the YY1 promoter was kindly provided by Dr. Denis C. Guttridge (Ohio State University). To construct the α-SMA-luciferase reporter plasmid, the α-SMA promoter was amplified by PCR using the primers (Forward 5’- CCCGCTCGAGATGGTCCTTAATCATGCT; Reverse 5’- CCCAAGCTTCTTACCCTGACAGCGACTGG). Amplification was completed using mouse lung fibroblast genomic DNA. The DNA was cut with *Xhol* and *HindIII* and subcloned into the *Xhol* and *HindIII* sites of the pGL3 basic vector (Promega). We transiently transfected the reporter constructs into MRC-5 cells using electroporation (300V 1050 Capacitance). These plasmids included YY1-Luc and α-SMA-Luc. The transfected cells were placed in OPTI-MEM media containing 0.1% FBS overnight in 24-well plates, followed by stimulation with IL-13 for 24 h. Using the same procedure, we cotransfected a YY1 plasmid with α-SMA-Luc.

### Lentiviral YY1 shRNA

YY1 shRNA constructs used for depletingYY1 were obtained from Open Biosystems (Huntsville, AL). For each gene, five pre-made constructs were obtained and tested. The constructs were used to identify genes capable of achieving efficient knockdown of YY1 at the protein level. Negative control constructs for the same vector system (vector alone, scrambled, and luciferase shRNA) were created in our lab. The lentiviral helper plasmids pHR'8.9ΔR and pCMV-VSV-G were obtained from Dr. Linzhao Chen (Johns Hopkins University). All plasmids were prepped, and their integrity was confirmed by restriction analysis. To prepare transient virus stocks, 1.0 x 10^6^ 293T cells were plated in 60-mm dishes. The next day, the cells were cotransfected with shRNA constructs (1.5 μg) along with pHR'8.9ΔR and pCMV-VSV-G helper constructs (1.5 μg and 0.3 μg, respectively) using FuGENE 6 (Roche, Indianapolis, IN); the medium was changed the following day. One day later, lentivirus-containing media was harvested. The viral stocks were centrifuged and filtered to remove any fragment in the medium. Next, LL97A cells were infected with shRNA lentiviruses. To accomplish this, cells were plated at subconfluent densities and infected one day later with a cocktail of 1 ml of virus-containing medium, 3 ml of regular medium, and 8 μg/ml of polybrene. The medium was changed one day after infection. Selective medium was added two days post-infection (2 μg/ml of puromycin for LL97A cells). After three days of puromycin selection, the mock-infected cells had all died. Stably-infected, pooled clones were then studied. shRNA sequences were as follows: Control-shRNA-1 sense: 5’ CCG GGC AGC TGC CAG ATA GCA TGA ACT CGA GTT CAT GCT ATC TGG CAG CTG CTT TTT G; Control-shRNA-1 antisense: 5’ AAT TCA AAA AGC AGC TGC CAG ATA GCA TGA ACT CGA GTT CAT GCT ATC TGG CAG CTG C. YY1-shRNA-1 sense: 5’CCG GGC CCT CAT AAA GGC TGC ACA ACT CGA GTT GTG CAG CCT TTA TGA GGG CTT TTT G; YY1 shRNA-1 antisense: CAA AAA GCC CTC ATA AAG GCT GCA CAA CTC GAG TTG TGC AGC CTT TAT GAG GGC CCG G.

### Western blot analysis

Cells were lyzed in lysis buffer (50 mM Tris/HCl (pH 8.0), 150 mM NaCl, 0.1% SDS, 0.5% sodium deoxycholate, 0.02% Na_3_N, 1% Nonidet P40, 1 mM PMSF and 2 μg/ml aprotinin). Protein concentrations were determined by BCA assay. Protein samples (20 μg) were separated by 8–16% SDS/PAGE gels and transferred onto a nitrocellulose membrane. The membrane was incubated with primary antibodies diluted in 1% non-fat milk in PBS containing 0.1% Tween 20, followed by incubation with horseradish peroxidase-conjugated IgG secondary antibody. Visualization was carried out using an enhanced chemiluminescence kit.

### Statistical analysis

Quantitative PCR, YY1, α-SMA promoter reporter assays were analyzed for statistical significance by using the paired Student’s t-test using Microsoft Excel software. p value of <0.05 was considered statistically significant. Western blot images were plotted by Image-J and were analyzed for statistical significance with Student’s t-test.

## Results

### IL-13 induces YY1, collagen and α-SMA expression in lung fibroblasts

**Fig 1:**
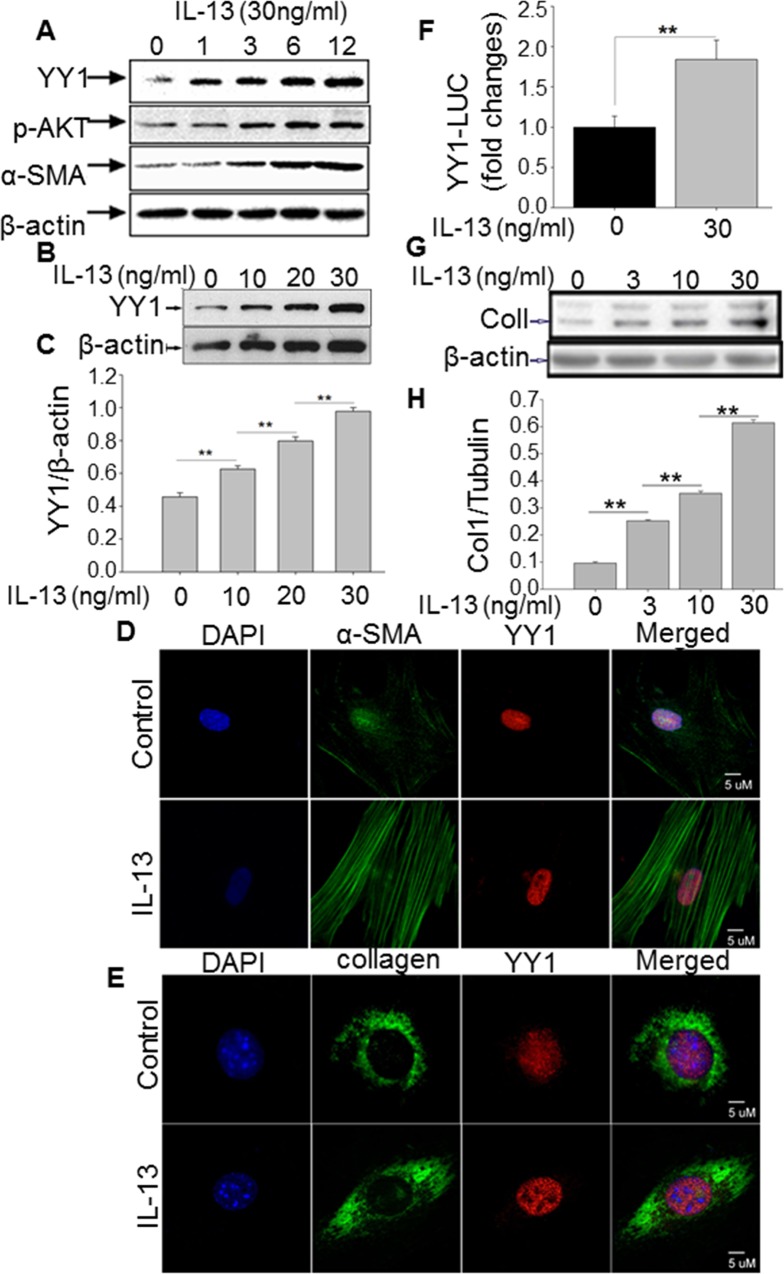
IL-13 induces YY1, collagen and α-SMA expression in lung fibroblasts. After 24 h serum starvation, lung fibroblasts (MRC5) were treated with 30 ng/ml of IL-13 in serum-free DMEM. (A) Expression of α-SMA, p-AKT and YY1 was determined by Western blot in MRC5 treated with IL-13 (30 ng/ml) for 0–12 h. Whole cell extracts were subjected to 8–16% SDS-polyacrylamide gel electrophoresis and immunoblotted with anti-YY1 and anti-α-SMA antibodies. (B) MRC5 cells were treated with IL-13 (10, 20, 30 ng/ml) for 12 h, and then the level of YY1 was determined by Western blot. (C) The image of western blot was scanned three times with ImageJ, and the density of YY1 was analyzed after normalization into β-actin. The statistic of scanned bands was performed by T-test. ** indicated P<0.01. (D and E) MRC5 cells were treated with IL-13 (30 ng/ml) for 24h, and immunostaining was performed to determine α-SMA (green), collagen I (green), YY1 (red), and DAPI (blue). All figures are at original magnification of 63×. Supplemental data S1 Fig. showed low magnification of Fig. 1D and 1E. (F) YY1-luciferase plasmid was transiently transfected into MRC5 cells by electroporation overnight followed by treatment with PBS or IL-13 (30 ng/ml) for 24 h. Relative light units (RLU) were measured with a luminometer. The fold change was calculated after normalization to control. The data was presented with standard errors derived from at least three independent experiments, each performed in triplicate. ** indicated P<0.01. (G) Fibroblasts were incubated in complete medium (MEM 10% FBS) for 24 h. On the second day the complete medium was replaced by medium with 0.1% FBS. Different doses of IL-13 (0, 3, 10 and 30 ng/ml) were added into the cells for 24 h. The cells were harvested, and collagen I in fibroblasts was measured by western blot in at least three independent experiments. (H) Densitometry of Western blot from Fig. 1G was analyzed by ImageJ. ** indicated P≤0.01.

To determine whether IL-13 induces YY1 expression in lung fibroblasts, human lung fibroblasts (MRC5) was cultured in a serum-free medium for 24 h. MRC5 cells were stimulated by IL-13 (30 ng/ml) for 1, 3, 6 and 12 h. We found that the levels of YY1, α-SMA and p-AKT were up-regulated in a time-dependent manner in IL-13-treated MRC5 as determined by Western blot (**Fig. 1A**). Furthermore, we incubated lung fibroblasts with different doses of IL-13, and found that YY1 expression was increased by IL-13 in a dose-dependent manner (**Fig. 1B and C**). These findings were confirmed by the immunofluorescent staining, which showed increased expression of α-SMA and collagen along with YY1 in MRC5 treated with IL-13 for 12 h (30 ng/ml) (**Figs. 1D** and **1E,** and **S1 Fig.**). In order to confirm the regulation of YY1 by IL-13, YY1-luciferase was transfected into MRC5 cells followed by treatment with IL-13 (30 ng/ml) for 24 h. We found that IL-13 treatment significantly upregulated YY1 promoter reporter activity in lung fibroblasts (**Fig. 1F**). We also measured collagen levels by Western blot following IL-13 treatment (0, 3, 10 and 30 ng/ml) (**Fig. 1G and 1H**). We found that collagen was significantly increased in a dose dependent manner. These data suggest that IL-13 induces YY1, p-AKT, collagen and α-SMA expression in lung fibroblasts.

### IL-13 induces α-SMA expression through YY1

**Fig 2:**
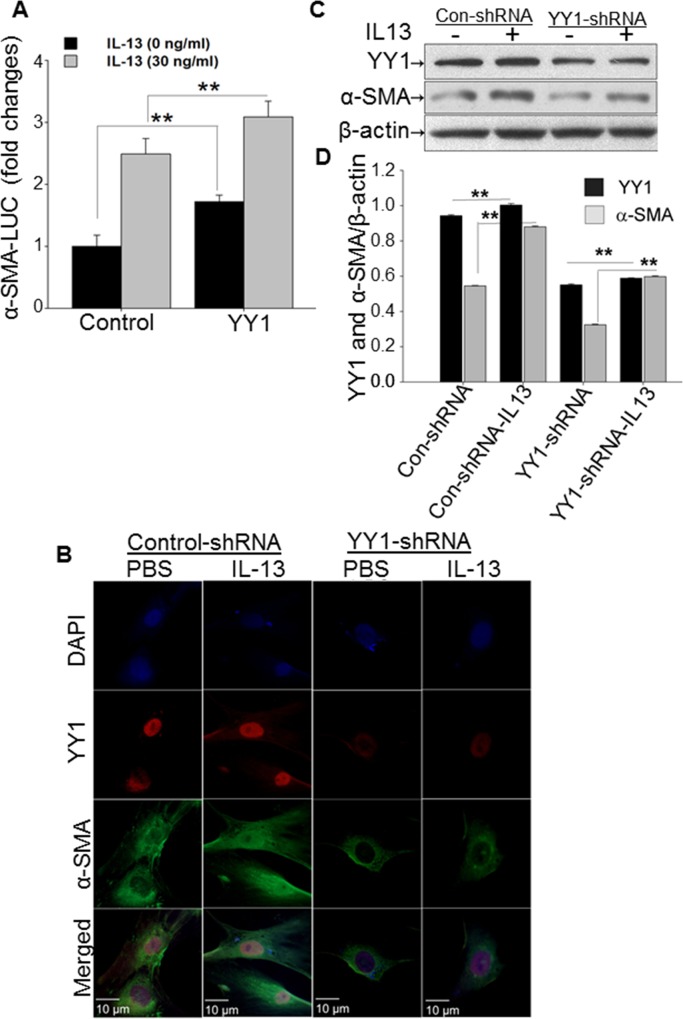
IL-13 induces α-SMA expression through YY1. (A) The α-SMA luciferase reporter (α-SMA-Luc, 5 μg) along with a 2.5 μg of control vector or 2.5 μg of YY1 was transiently transfected by electroporation into MRC-5 cells. At 24 h after transfection, IL-13 (30 ng/ml) or PBS were used to treat the MRC-5 cells for 24 h. These cells were lyzed with luciferase lysis buffer. Relative light units (RLU) were measured with a luminometer. The data are presented with standard errors derived from at least three independent experiments, mean ± SEM. **P < 0.01. (B) LL97A cells were infected with μl of control lentivirus vector shRNA (1x10^5^) or 10μl of lentivirus vector YY1 shRNA (1x10^5^) for 2 days. Lentiviral particles were produced as described in our previous paper [23]. Puromycin (4 μg/ml) was then used to select the transduced cells, which were stimulated by IL-13 (30 ng/ml) for 24 h. The selected LL97A cells were immunostained with anti-α-SMA (green) and anti-YY1 (red) antibodies. Representative pictures are presented at the original magnification shown a ruler on image. Representative examples are from three independent experiments. (C) The levels of YY1 and α-SMA in LL97A cells transduced and stimulated with IL-13 (30 ng/ml) were determined by western blot using corresponding antibodies. (D) Densitometry of western blot from 2C is measured by ImageJ. ** indicated p≤0.01.

Increased α-SMA expression is an important characteristic of fibroblast activation and the myofibroblast phenotype. We have previously demonstrated that YY1 regulates α-SMA by directly binding its promoter as determined by the electrophoretic mobility shift assay and chromatin immunoprecipitation approaches [23]. This suggests that IL-13 may regulate α-SMA though regulation of YY1 in lung fibroblasts. To test this concept, we constructed and transfected an α-SMA promoter driven luciferase plasmid into fibroblasts along with an YY1 plasmid. We found that α-SMA reporter activity was significantly upregulated by overexpressing YY1 in lung fibroblasts, and this effect was further increased by IL-13 treatment (30 ng/ml) (**Fig. 2A**). In order to further determine if YY1 mediates IL-13-induced α-SMA expression, YY1 was knocked down in human lung fibroblasts (LL97A) by transduction of an YY1 lenti-shRNA construct. We found that α-SMA expression was reduced in fibroblasts by knockdown of YY1 with and without IL-13 treatment (30 ng/ml) compared to cells treated with control shRNA (**Fig. 2B**). Similarly, the level of α-SMA was decreased in LL97A cells transducted with an YY1 lenti-shRNA construct by Western blot (**Fig. 2C and 2D**). These data suggest that IL-13-induced α-SMA expression is at least, partially mediated by YY1 in lung fibroblasts.

### YY1 is up-regulated in the lung fibroblasts of IL-13 overexpressing/transgenic mice

**Fig 3:**
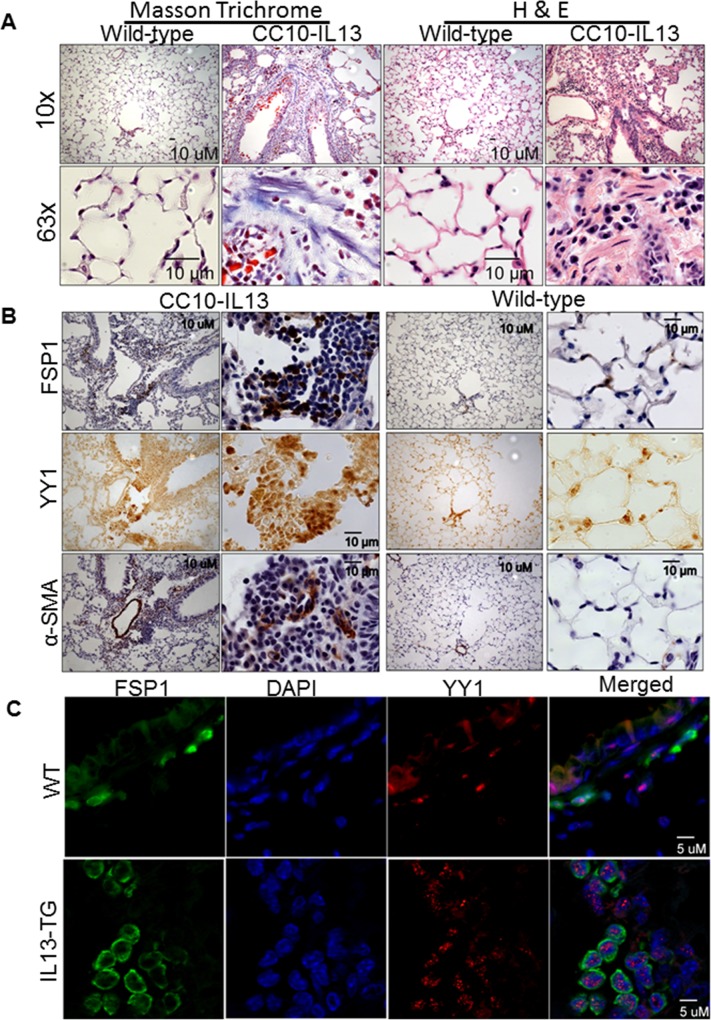
Fibroblast specific protein 1 (FSP1), α-SMA, YY1, and collagen were increased in CC10-IL-13 transgenic mice. **(A)** Lung tissues were stained with Hematoxylin and eoxin and Masson trichrome. The histological features and collagen were compared in between wild type (WT) control mice and CC10-IL-13 transgenic mice. All figures are at original magnification with a ruler (upper panel) and with a ruler (lower panel) on the images. **(B)** Immunohistochemical staining was used to localize the sites of FSP1, α-SMA, and YY1 in lungs of CC10-IL-13 transgenic mice and WT l mice. Collagen stains blue in these panels. All figures are at original magnification: 10 × (left panel) and 63 × (right panel). YY1, FSP1, and α-SMA expression are increased in lung tissue from IL-13 transgenic mice (n = 4). **(C)** Lung tissues from IL-13 transgenic (IL-13tg) and wild-type mice were stained by immunofluorescence for YY1 (Red), FSP1 (Green) and DAPI (blue). The magnification is 63x. Low magnification 10x showed in supplemental data S1 Fig.

To test if YY1 is regulated in fibroblasts by IL-13 *in vivo*, we used the transgenic mice in which IL-13 is overexpressed in lung Clara cells (CC10-IL-13) [27]. YY1 was driven by the CC10 promoter. In agreement with our previous findings [27], the expression of collagen was increased in CC10-IL-13 mice by masson trichrome staining (**Fig. 3A**). In addition, lung fibrosis was evident in CC10-IL-13 transgenic mice, but not in wild-type mice via H&E staining (**Fig. 3A**). Importantly, YY1 was up-regulated along with increased expression of both FSP1 (a marker of fibroblasts) as well as other cells, and α-SMA in lungs of CC10-IL-13 mice using immunohistochemistry (**Fig. 3B**). Although we have previously shown that YY1 is co-localized in lung fibroblasts in IPF patients [23], it is unclear if YY1 is overexpressed in lung fibroblasts of IL-13 transgenic mice. After double stained of YY1 and fibroblasts with corresponding anti-YY1 and anti-FSP antibodies, we found that YY1 expression was located and increased in lung fibroblasts of CC10-IL-13 mice (**Fig. 3C**). These data suggest that YY1 is up-regulated by IL-13 in lung fibroblasts *in vivo*. In addition, we also noted increased YY1 staining in AT2 cells and bronchial epithelial cells.

### IL-13-induced YY1 expression is regulated by AKT and collagen and α-SMA were regulated by p-AKT inhibitor

**Fig 4:**
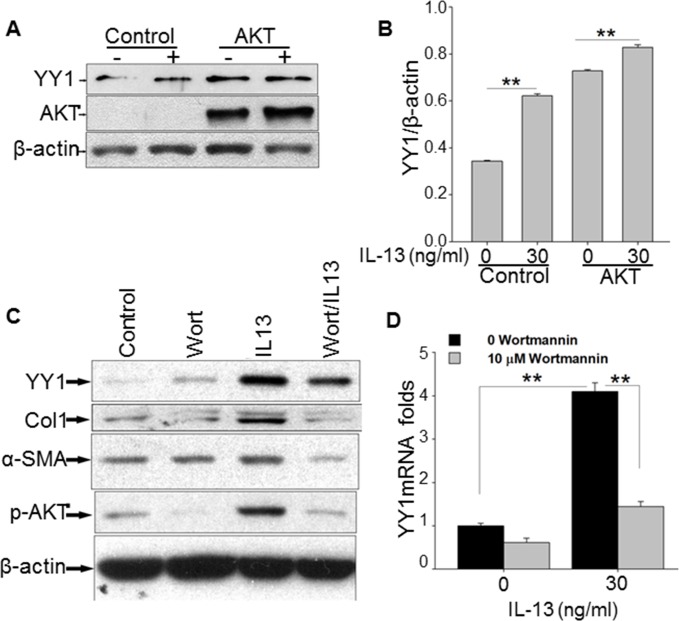
IL-13-induced YY1 expression is regulated by AKT pathway. MRC-5 cells were transfected with AKT and pCDNA1 control plasmids with electroporation. (A). At 24 h after transfection, the cells were starved for 24 h. IL-13 (30 ng/ml) was added to the cells for 12 h, The cells were lyzed, and the levels of YY1, AKT, p-AKT and β-actin were determined by Western blot. (B). YY1 and β-actin expression from Fig. 4A were scanned and were conducted a densitometric analysis with Image J. YY1 expression was normalized to β-actin. Scanned data were analyzed by T-test. ** indicate p value <0.01. (C) After MRC-5 cells were starved for 24 h without serum, cells were pre-treated with or without wortmannin (10 μM) for 1 h, and then treated with or without IL-13 (30 ng/ml) for 12 h in serum-free DMEM. Whole cell extracts were subjected to Western blot analysis for determining the levels of YY1, α-SMA, p-AKT, collagen and β-actin. (D). the mRNA expression of YY1 was detected by quantitative PCR. GAPDH mRNA expression was used as an internal control. The data are presented with standard errors derived from at least three independent experiments, each performed in triplicate; n = 3 and **p < 0.01.

We and others have shown that IL-13-induced lung fibrosis is associated with activation of AKT and YY1 signaling [11]. However, it remains unclear if IL-13 induces YY1 expression through AKT activation. In order to answer this question, we transfected an AKT plasmid in MRC5 cells following by the treatment of IL-13 (30 ng/ml) for 24 h. We found that YY1 was up-regulated in the fibroblasts transfected with the AKT plasmid compared to that with control plasmid (pcDNA1), which was further augmented by IL-13 treatment (**Fig. 4A and 4B**). Treatment with an AKT phosphorylation inhibitor (wortmannin, 5 μM) decreased the level of YY1 induced by IL-13 in MRC5 cells determined by Western blot (**Fig. 4C**). Similarly, AKT inhibitor treatment suppressed IL-13-induced increase of α-SMA and collagen (**Fig. 4C**). Using quantitative PCR, we also found that YY1 mRNA was decreases by wortmannin in lung fibroblasts (**Fig. 4D**). These data suggest that IL-13 induces YY1 expression through AKT activation in lung fibroblasts.

### AKT phosphorylation is increased in lung of CC10-IL-13 mice

**Fig 5:**
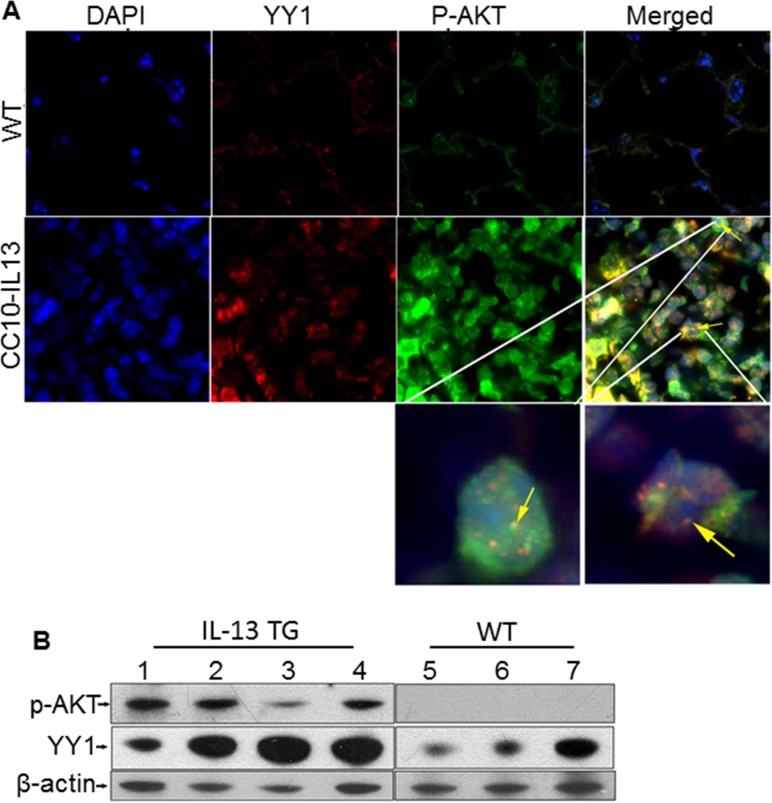
YY1 and p-AKT were up-regulated in the lungs of IL-13 transgenic mice. (A) Lungs from IL-13 transgenic mice and wild type (WT) mice were perfused with PBS, fixed with 10% formaldehyde, and embedded in paraffin. Immunofluorescent staining was conducted with anti-YY1 (green) and anti-p-AKT (red). DAPI is blue. The pictures are presented at the original magnification showed as ruler on the image. Representative examples are from three independent experiments. In a big magnification image, yellow arrows indicate YY1 and p-AKT overexpressed in the same area. (B) A portion of lung was homogenized, and Western blot was performed to determine the levels of YY1 and p-AKT in both IL-13 transgenic and wild-type mice.

AKT phosphorylation occurs in the lungs of IPF patients and in fibrotic animal models [28,29,30]. It is unclear if IL-13-induced fibrosis is associated with AKT pathway activation and YY1 expression *in vivo*. Therefore, we determined whether AKT phosphorylation (p-AKT) is upregulated by IL-13 overexpression in CC10-IL-13 transgenic mouse. Both immunofluorescent staining and Western blot were performed to determine the expression of YY1 and p-AKT in lungs of both CC10-IL-13 transgenic and wild-type mice. As expected, both p-AKT (Ser473) and YY1 were increased in the fibrotic areas (**Fig. 5A**) and in whole lung tissue of CC-10-IL-13 mice as compared to wild-type mice (**5B**). These data suggest that IL-13 up-regulates AKT phosphorylation and YY1 expression *in vivo*.

## Discussion

IPF is characterized by varying degrees of interstitial fibrosis. IL-13 is a strong inducer of tissue fibrosis, and activates survival pathways [31]. IL-13 is also known to regulate α-SMA expression in fibroblasts and to promote the myofibroblast phenotype [32]. Both YY1 and AKT are involved in cell survival as well as in lung fibrosis [11,23]. However, it is unclear whether YY1 is an essential mediator of IL-13-induced lung fibrosis. We, therefore, hypothesized that YY1 is induced by IL-13, which plays a critical role in the pathogenesis of fibrotic responses in the lung. In this study we have found that increasing YY1 enhances, whereas decreasing YY1 reduces fibroblast differentiation induced by IL-13. Furthermore, we have demonstrated that IL-13–induced YY1 expression is mediated by AKT pathway activation in fibroblasts *in vitro*. Correspondingly, *in vivo* in IL-13 overexpressing mice both YY1 and AKT are activated.

It has been reported that IL-13 induces lung fibrosis, and IL-13 antagonist have been tested in lung fibrosis and other lung diseases [33]. NeoPharm announced a FDA orphan drug designation for IL13-PE38QQR for treating IPF in 2010 [34]. IL-13 can bind to cell membrane receptors, IL-4 receptor-1, IL-13 receptor α1 and receptor α2 which is located in chromosome Xq24 [15] in fibroblasts. Recent reports have demonstrated that IL-13 regulates TGF-β through IL-13 Rα2 and AP1 (Activate Protein) pathways [17]. Although IL-13 regulates several pathways, such as Stat6, PI3K-AKT and NF-κB, there is no study regarding the regulation of YY1 expression by IL-13 and the underlying mechanisms. In the study we first demonstrated that YY1 is involved in fibroblast differentiation by IL-13. The regulation is mediated by p-AKT.

In supplemental data, we found that C-jun was upregulated by IL-13 (**S3A Fig.**). We showed that YY1 reporter was upregulated by AP1 (cjun/cfos) in lung fibroblasts (**S3B Fig.**). This suggests that YY1 is regulated by AP1. Although AP1 regulates YY1, we do not know whether AP1 is regulated by IL-13Rα1 or IL13Rα2. We showed that IL-13Rα1 and IL-13Rα2 expression were upregulated in lung fibroblasts by IL-13 (**S3C Fig.**), which suggests either, may be involved. Further studies are ongoing to define the role of each receptor.

In our previous publication, we demonstrated that YY1 can regulate collagen and α-SMA expression in lung fibroblasts by directly binding their promoters, and decreasing YY1 significantly decreased collagen and α-SMA expression [23]. IL-13 has been shown to increase α-SMA expression, and to cause myofibroblastic differentiation [35]. Both α-SMA expression and type-I collagen are significantly inhibited by pre-treatment with an AKT inhibitor, LY294002, in lung fibroblasts [36], and inhibitors of PI3K/AKT pathway (LY294002 and wortmannin) can reduce lung fibrosis *in vivo* [37]. In the study for the first time, we show that IL-13-induced fibroblast differentiation is mediated by YY1. Furthermore, AKT activation is involved in the pathway by which IL-13 induces fibroblast differentiation mediated by YY1. Inhibiting AKT by its inhibitor, wortmannin reduced YY1, p-AKT and α-SMA by induced by IL-13 in lung fibroblasts (**Fig. 4C**).

YY1 is a nuclear factor essential for mammalian development, and plays important roles in cell differentiation [38]. It has been found that IL-13 activates fibroblast differentiation through AKT pathway.[11,39]. In this study we have first found that YY1 was induced in lung fibroblasts by AKT pathway activation. Although we do not know why increasing AKT protein increases YY1 expression as well as α-SMA, we predict that the AKT is phosphorylated by an endogenous kinase in fibroblasts. In Fig. 4A of AKT transfection assay we noticed that the signal and level of endogenous AKT in control transfection group is weak. This is due to the short-term exposure of membranes/blots to X-ray films, which avoids the over-thick bands in cells after transfection of constitutive AKT plasmid with high overexpression of AKT. Nevertheless, we provided an image with a longer time exposition in supplement data which showed a small amount of endogenous AKT in the fibroblasts (**S2 Fig.**).

Wortmannin has been shown to inhibit PKB/AKT phosphorylation in a time- and dose-dependent manner and inhibits AKT Ser-473 phosphorylation [40]. Using the Akt inhibitor, we demonstrated that YY1 and p-AKT are inhibited in lung fibroblasts, and fibrotic markers, collagen and α-SMA are decreased (**Fig. 4C**). Conversely, overexpressing AKT increases YY1 expression in fibroblasts (**Fig. 4A**). This suggests that AKT signaling modulates YY1 expression. We found that YY1 is decreased by wortmannin in lung fibroblasts regardless of IL-13 stimulation. This implies that phosphorylated AKT plays a role in increasing YY1 expression. Importantly, *in vivo*, phosphorylated AKT and YY1 were increased in lungs of IL-13 transgenic mice (**Fig. 5A and 5B**). This provides additional evidence that increasing phosphorylated AKT is important in inducing upregulation of YY1 expression during IL-13-induced fibrosis.

We also demonstrated that IL-13 induced α-SMA, which was mediated by YY1 and AKT signaling. This suggests that fibroblast differentiation is regulated by AKT pathway. We have previously demonstrated that YY1 regulates α-SMA expression by directly binding to its promoter [23]. This suggests that IL-13-induced α-SMA expression may be through activated YY1 and phosphorylated AKT. Fig. 2 showed that α-SMA was regulated by the variant YY1 in fibroblasts. Decreasing phosphorylation of AKT not only decreases α-SMA expression but also inhibits YY1 expression. This demonstrated that IL-13 regulates fibroblast differentiation through activated YY1 and phosphorylated AKT. Interestingly, the expression of α-SMA, YY1 and phosphorylated AKT are increased in IL-13 transgenic mice. We also observed YY1 expression in FSP1 positive fibroblasts may, but not all of these cells are fibroblasts. These data demonstrated that accumulation of fibroblasts during lung fibrosis induced by overexpressing IL-13 is associated with activation of AKT pathway and induction of YY1 expression. Further study is required to further determine the casual relationship of IL-13-YY1 and AKT signaling *in vivo*. This will be achieved by generating crossed IL-13 transgenic and YY1 KO mice, and by assessing if AKT can reduce fibrogenesis challenge in IL-13 deficient mice.

This study does not imply that mouse and human tissue will be identical. However, overexpressing IL-13 in mouse lung help us to understand the mechanism of IL-13 regulates fibrotic genes in lung fibroblasts. It is unlikely at the current time that a similar analysis in human samples will be performed, although an analysis using non-human primates is feasible. For the majority of fibrotic gene conserved between mouse and human, their developmental pattern of expression may be similar.

In summary, this study demonstrates that YY1 is a critical effecter in IL-13-induced fibroblast differentiation, and we highlight an important role for AKT signaling in lung fibroblast differentiation. Targeting YY1 and AKT along with IL-13 would provide a promising avenue in intervening the progress and development of lung fibroblast differentiation.

## Supporting Information

S1 Fig (PDF)

S2 Fig (PDF)

S3 Fig (PDF)

S1 Method (DOCX)
