# Supplementary material for: IL-13 Induces YY1 through the AKT Pathway in Lung Fibroblasts
Source: PLoS One. 2015 Mar 16;10(3):e0119039. doi: 10.1371/journal.pone.0119039 (PMC4361578; doi:10.1371/journal.pone.0119039)
Supplement: S1 Fig — (PDF) [file pone.0119039.s001.pdf]

This is the S1 Fig.

S1 Fig. is low magnification (10x) of Figs. 1D and E.

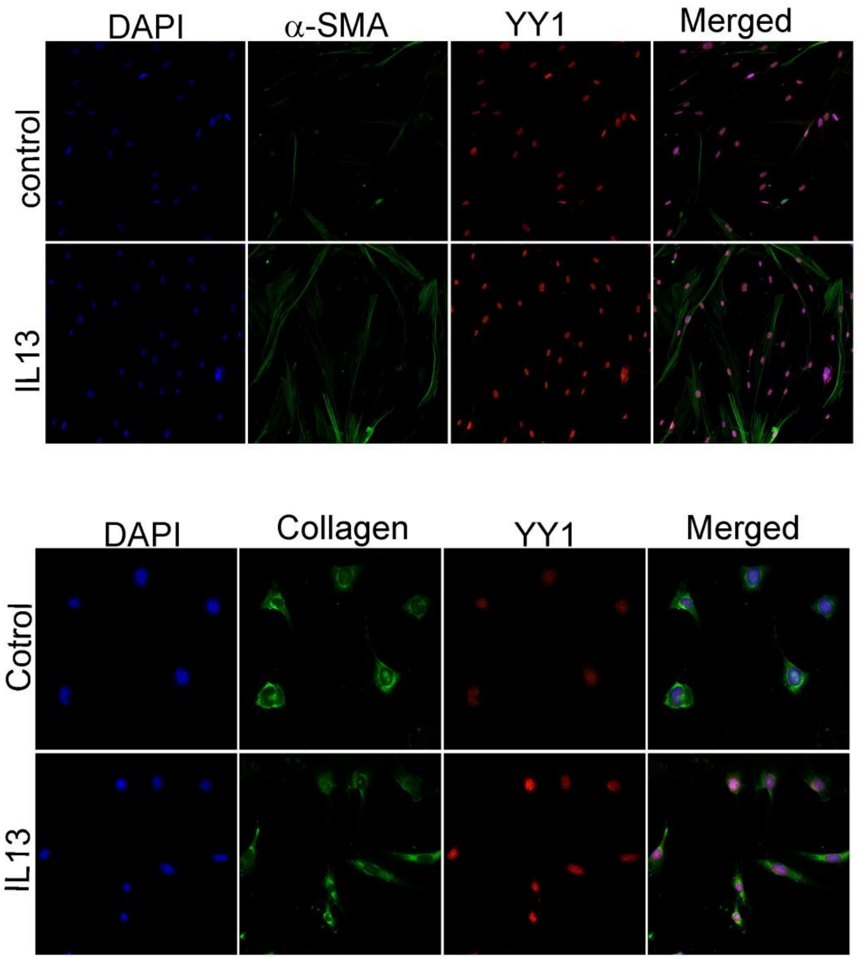

S1 Fig.
MRC5 cells were treated with IL-13 (30 ng/ml) for 24h, and immunofluorescent staining was performed to determine α-SMA (green), collagen I (green), YY1 (red), and DAPI (blue). All figures are at original magnification of 10×.
