# Supplementary material for: IL-13 Induces YY1 through the AKT Pathway in Lung Fibroblasts
Source: PLoS One. 2015 Mar 16;10(3):e0119039. doi: 10.1371/journal.pone.0119039 (PMC4361578; doi:10.1371/journal.pone.0119039)
Supplement: S2 Fig — (PDF) [file pone.0119039.s002.pdf]

This is the S2. Fig. Title. Endogenous AKT was expressed in fibroblasts determined by western blot with a long time exposition.

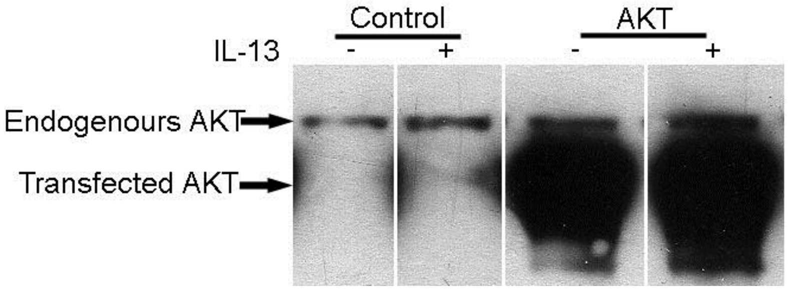

S2 Fig.
MRC-5 cells were transfected with AKT and pCDNA1 control plasmids with electroporation**.** (A). At 24 h after transfection, the cells were starved for 24 h. IL-13 (30 ng/ml) was added to the cells for 12 h, The cells were lyzed, and the levels of AKT was determined by Western blot. The blotting membrane was exposed to x-ray film 3 times than Fig. 4A.
