# Supplementary material for: IL-13 Induces YY1 through the AKT Pathway in Lung Fibroblasts
Source: PLoS One. 2015 Mar 16;10(3):e0119039. doi: 10.1371/journal.pone.0119039 (PMC4361578; doi:10.1371/journal.pone.0119039)
Supplement: S3 Fig — (PDF) [file pone.0119039.s003.pdf]

This is the S3 Figs.

c-Jun was upregulated by IL13 (S3A.Fig) and YY1 reporter was upregulated by cotransfected with c-jun and c-fos (S3B.Fig). mRNA of IL13Rα1 and IL13Rα2 were upregulated by IL13 in lung fibroblasts (S3C.Fig).

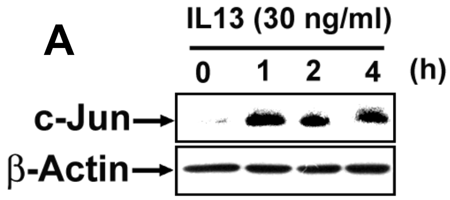

S3A Fig.

Fibroblasts were incubated in serum free medium. On day 2, the cells were stimulated by IL13 (30ng/ml) for different time points. C-Jun and b-actin were determined by western blot.

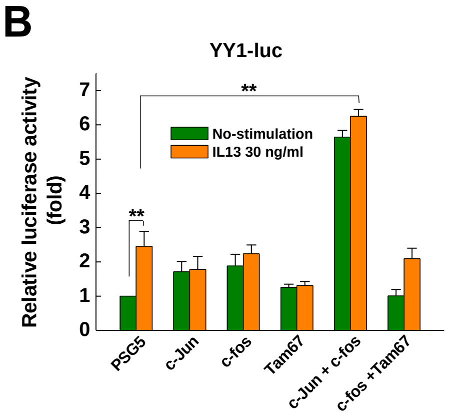

S3B Fig.

Plasmids, c-jun, c-fos, Tam67 (dominant negative c-fos) and YY1-luc reporter were transfected or cotransfected into fibroblasts by electroporation respectively. After transfection, the cells were stimulated with IL13 overnight. The YY1 reporter was significantly upregulated by co-transfected with cJun/c-fos. The activity was inhibited by dominant negative c-fos. This suggests that YY1 is regulated by AP1 in lung fibroblasts.

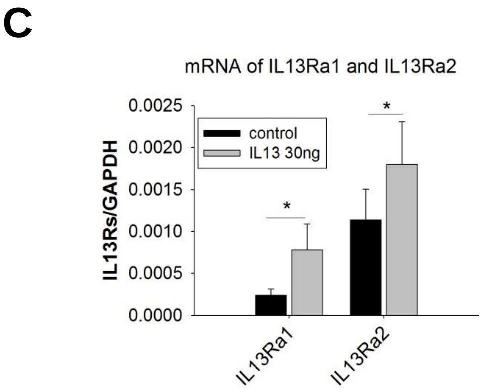

S3C Fig.

Fibroblasts (MRC5) were incubated in serum free medium for 24 hours and were stimulated by IL13 (30ng/ml) for 4 hours. mRNA was extracted from these cells by Trizol. After reverse transcription, a real time PCR was performed. PCR reaction cycle was converted by formula $2^{-\Delta(\text{gene-control})}$.
