# Supplementary material for: IL-13 Induces YY1 through the AKT Pathway in Lung Fibroblasts
Source: PLoS One. 2015 Mar 16;10(3):e0119039. doi: 10.1371/journal.pone.0119039 (PMC4361578; doi:10.1371/journal.pone.0119039)
Supplement: S1 Method — (DOCX) [file pone.0119039.s004.docx]

**Supplemental data**

**Method**

**Lentiviral YY1 shRNA**

YY1 shRNA constructs used for depleting YY1 were obtained from Open Biosystems (Huntsville, AL). For each gene, five pre-made constructs were obtained and tested. The constructs were used to identify genes capable of achieving efficient knockdown of YY1 at the protein level. Negative control constructs for the same vector system (vector alone, scrambled, and luciferase shRNA) were created in our lab. The lentiviral helper plasmids pHR'8.9
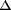
R and pCMV-VSV-G were obtained from Dr. Linzhao Chen (Johns Hopkins University). All plasmids were prepped, and their integrity was confirmed by restriction analysis. To prepare transient virus stocks, 293T cells (1.0 x 10^6^ cells) were plated in 60-mm dishes. The next day, the cells were cotransfected with shRNA constructs (1.5 µg) along with pHR'8.9
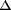
R and pCMV-VSV-G helper constructs (1.5 µg and 0.3 µg, respectively) using FuGENE 6 (Roche, Indianapolis, IN); the medium was changed the following day. One day later, lentivirus-containing media was harvested. The viral stocks were centrifuged and filtered to remove any fragment in the medium. Next, LL97A cells were infected with shRNA lentiviruses. To accomplish this, cells were plated at subconfluent densities and infected one day later with a cocktail of 1 ml of virus-containing medium, 3 ml of regular medium, and 8 µg/ml of Polybrene. The medium was changed one day after infection. Selective medium was added two days post-infection (2 µg/ml of puromycin for LL97A cells). After three days of puromycin selection, the mock-infected cells had all died. Stably-infected, pooled clones were then studied. shRNA sequences were as follows:

Sequence of shRNA hirepin.

Control-shRNA-1 sense:5’ CCGGGCAGCTGCCAGATAGCATGAACTCGAGTTCATGCTATCTGGCAGCTGCTTTTTG Control-shRNA-1 antisense:5’ AATTCAAAAAGCAGCTGCCAGATAGCATGAACTCGAGTTCATGCTATCTGGCAGCTGC YY1-shRNA-1 sense 5’CCGGGCCCTCATAAAGGCTGCACAACTCGAGTTGTGCAGCCTTTATGAGGGCTTTTTG YY1 shRNA-1 antisense CAAAAAGCCCTCATAAAGGCTGCACAACTCGAGTTGTGCAGCCTTTATGAGGGCCCGG**.**

Realtime PCR primers

The sequences of the YY1 primers were listed (Forward 5’- ACGCTGGTCACCGTGGCGGC; reverse 5’- TTGCCGCTCTTCTTGCCGCC and GAPDH: Forward 5’-GAAGGCCATGCCAGTGAGCT reverse 5’- GCCACCCAGAAGACTGTGGAT).

α-SMA luciferase construct

(Forward 5’- CCCGCTCGAGATGGTCCTTAATCATGCT; Reverse 5’- CCCAAGCTTCTTACCCTGACAGCGACTGG).

Western blot

Cells were lysed in a lysis buffer (50 mM Tris/HCl (pH 8.0), 150 mM NaCl, 0.1% SDS, 0.5% sodium deoxycholate, 0.02% Na_3_N, 1% Nonidet P40, 1 mM PMSF and 2 *µ*g/ml aprotinin). Protein concentrations were determined by BCA assay. Protein samples (20 *µ*g) were separated by 8-16% SDS/PAGE gels and transferred onto a nitrocellulose membrane. The membrane was incubated with primary antibodies diluted in 1% non-fat milk in a PBS containing 0.1% Tween 20, followed by incubation with a horseradish peroxidase-conjugated IgG secondary antibody. Visualization was carried out using an ECL (enhanced chemiluminescence) kit.

Plasmid containing only the YY1 promoter was kindly provided by Dr. Denis C. Guttridge (Ohio State University). To construct the α-SMA-luciferase reporter plasmid, the α-SMA promoter was amplified by PCR using the primers Amplification was completed using mouse lung fibroblast genomic DNA. The DNA was cut with *Xhol* and *HindIII,* and subcloned into the *Xhol* and *HindIII* sites of the pGL3 basic vector (Promega). We transiently transfected YY1-luc or the α-SMA-Luc reporter constructs into MRC5 cells using electroporation (300V, 1050 Capacitance). The transfected cells were placed in OPTI-MEM media containing 0.1% FBS overnight in 24-well plates, followed by stimulation with IL-13 for 24 h. Using the same procedure, we cotransfected a YY1 plasmid with α-SMA-Luc.
